# Relationship of cytochrome P450 gene polymorphisms with blood concentrations of hydroxychloroquine and its metabolites and adverse drug reactions

**DOI:** 10.1186/s12920-022-01171-6

**Published:** 2022-02-08

**Authors:** Beibei Gao, Tingfei Tan, Xi Cao, Menglu Pan, Chunlan Yang, Jianxiong Wang, Zongwen Shuai, Quan Xia

**Affiliations:** 1 Department of Pharmacy, The First Affiliated Hospital of Anhui Medical University, Hefei, China; 2 Department of Rheumatism and Immunity, The First Affiliated Hospital of Anhui Medical University, Hefei, China; 3 Inflammation and Immune Mediated Diseases Laboratory of Anhui Province, Hefei, China; 4 The Grade 3 Pharmaceutical Chemistry Laboratory of State Administration of Traditional Chinese Medicine, Hefei, China

**Keywords:** Hydroxychloroquine, CYP450 gene polymorphism, Blood concentration, Adverse drug reactions, Systemic lupus erythematosus, Rheumatoid arthritis

## Abstract

**Background:**

Hydroxychloroquine (HCQ) is a cornerstone therapy for systemic lupus erythematosus (SLE) and rheumatoid arthritis (RA). This study aimed to investigate the relationship of cytochrome P450 (CYP450) gene polymorphisms with blood concentrations of HCQ and its metabolites and adverse drug reactions (ADRs) in patients with SLE and RA.

**Methods:**

A cohort of 146 patients with SLE and RA treated with HCQ was reviewed. The ADRs of the patients were recorded. The blood concentrations of HCQ and its metabolites were measured by liquid chromatography-tandem mass spectrometry (LC-MS/MS) analysis. Genotyping of single nucleotide polymorphisms (SNPs) in CYP450, a metabolic enzyme involved in the HCQ metabolic pathway, was performed using a MassARRAY system. The chi-square test, T-test, and one-way analysis of variance were used to analyse data.

**Results:**

Among 29 candidate SNPs, we found that CYP3A4 (rs3735451) was significantly associated with blood levels of HCQ and its metabolites in both the unadjusted model and adjusted model (patients taking HCQ for > 10 years) (*P* < 0.05). For CYP3A5 (rs776746), a greater risk of skin and mucous membrane ADRs was associated with the TT genotype than with the CT + CC genotypes (*P* = 0.033). For CYP2C8 (rs1058932), the AG genotype carried a greater risk of abnormal renal function than the AA + GG genotype (*P* = 0.017); for rs10882526, the GG genotype carried a greater risk of ophthalmic ADRs than the AA + AG genotypes (*P* = 0.026).

**Conclusions:**

The CYP2C8 (rs1058932 and rs10882526) and CYP3A5 (rs776746) polymorphisms are likely involved in the ADRs of HCQ. Gene polymorphism analysis of CYP450 and therapeutic drug monitoring of HCQ and its metabolites might be useful to optimise HCQ administration and predict ADRs.

## Introduction

Autoimmune diseases such as systemic lupus erythematosus (SLE) and rheumatoid arthritis (RA) are caused by immune tolerance deficiency or abnormal immune regulation and lead to damage of the host organs [1]. Glucocorticoids (GCs) and disease-modifying anti-rheumatic drugs (DMARDs) are routinely prescribed treatments that have shown good therapeutic effects on disease control [2]. Currently, non-biological DMARDs like azathioprine, methotrexate (MTX), and hydroxychloroquine (HCQ) play a role in relieving pain and inhibiting disease progression [3]. Among these drugs, HCQ was initially used as an antimalarial medication and then translated to rheumatic diseases. Currently, HCQ is the mainstay treatment for SLE; according to the latest European guidelines, it is recommended for all SLE patients unless contraindicated or with adverse effects [4]. The latest European League Against Rheumatism (EULAR) recommendations stated that HCQ was also part of the triple therapy in RA, where it is combined with methotrexate and sulfasalazine to increase the response rate [5]. Despite the efficacy of HCQ in treating manifestations of SLE and RA, common side effects such as headaches, dizziness, gastrointestinal symptoms, and rash have been reported [6]. Notably, retinopathy is a serious side effect of HCQ, and regular ophthalmologic monitoring is recommended for patients on long-term HCQ therapy [7, 8].

HCQ shows large interindividual variations in its blood concentration, despite individuals taking the same dose. For SLE patients on long-term oral HCQ treatment, a lower SLE disease activity index (SLEDAI) score was significantly associated with higher blood HCQ concentration [9]. Moreover, metabolites of HCQ such as desethyl hydroxychloroquine (DHCQ) and desethyl chloroquine (DCQ) exhibited a concentration-dependent relationship in patients with RA; bisdesethyl chloroquine (BDCQ) has also been implicated in HCQ toxicity [10]. HCQ is metabolized into DHCQ, DCQ, and BDCQ by cytochrome P450 enzymes (CYP450s) 3A4/5, 2C8, and 2D6 in vivo by *N*-deethylation [11], in which DHCQ is the major metabolite and the activated form of HCQ. The CYP 2D6*10 (rs1065852) polymorphism is significantly associated with the level of DHCQ, while CYP3A5*3 (rs776746) and CYP 3A4*18 (rs28371759) did not show any significant association with the levels of HCQ and DHCQ [12]. However, to our knowledge, few studies thus far have reported the relationship between the adverse drug reactions (ADRs) of HCQ and CYP450 polymorphisms [13]. Understanding this relationship will be helpful to refine HCQ dosage. Therefore, we analysed the influence of CYP450 gene polymorphisms, mainly the 2D6, 3A4, 3A5, and 2C8 polymorphisms, on the blood concentrations of HCQ and its metabolites, as well as the risk of ADRs.

## Patients and methods

### Study design and population

This was a prospective, observational, single-centre clinical trial designed to examine the influence of CYP450 gene polymorphisms on the blood concentrations and ADRs of HCQ and its metabolites. The study subjects were from the Outpatient Department of Rheumatology and Immunology at the First Affiliated Hospital of Anhui Medical University and who had been taking long-term oral HCQ. The patients’ general and clinical details were recorded in detail. The inclusion criteria for SLE and RA were as follows: patients met either the 2010 RA classification criteria defined by the American College of Rheumatology (ACR) and the EULAR, or the EULAR/ACR-2019 for SLE [14, 15]; received treatment with oral HCQ for > 6 months; were on a daily dosage of 200–400 mg; and consented to donate blood samples for the study. The exclusion criteria were as follows: non-SLE and non-RA patients; pregnant and lactating women; patients with incomplete data; patients with poor compliance; and those with renal impairments, eye disease, or cutaneous damage before receiving HCQ. Blood samples (10 mL) were collected during an outpatient visit for determining HCQ, DHCQ, DCQ, and BDCQ blood concentrations and genetic testing. The following laboratory tests were conducted: complete blood cell count, erythrocyte sedimentation rate, C-reactive protein level, levels of C3 and C4, and anti-double-stranded DNA antibody titre.

### LC–MS/MS analysis of HCQ, DHCQ, DCQ, and BDCQ blood levels

We measured the blood concentrations of HCQ, DHCQ, DCQ, and BDCQ at Anhui Medical University Scientific Research Experiment Center (Anhui Medical University, Hefei City, China) with Liquid chromatography-tandem mass spectrometry (LC–MS/MS), according to the method of Chhonker et al. [16], by using an AB Sciex 5500 LC-30AD pump SIL-30AC autosampler (AB SCIEX, Los Angeles, CA, USA). The analytes were separated on a Poroshell 120 EC-C18 (2.1 × 100 mm, 2.7-μm thickness, Agilent Technologies, CA, USA), at a column temperature of 35 °C and flow rate of 0.25 mL/min. The internal standard for the LC–MS/MS assay was quinine. The MS data of blood separated by the above-mentioned optimised high-performance liquid chromatography (HPLC) method were processed using the Analyst 1.6.3 data processing software system (AB SCIEX, Los Angeles, CA, USA). According to the internal standard method, the standard curve was established in order to calculate the blood levels of HCQ, DHCQ, DCQ, and BDCQ.

### Evaluation of ADRs

The ADRs evaluation form for SLE and RA patients on HCQ was designed according to the World Health Organization-Uppsala Monitoring Centre (WHO-UMC) system [17] and recorded in our study. Patients with serious side effects were assigned to a specialist clinic for further treatment and regular follow-up until symptoms were stable.

### DNA extraction and genotyping of CYP SNPs

Genomic DNA was extracted using a commercially available DNA extraction kit (Tiangen Biotech, Beijing, China) according to the manufacturer’s instructions. The extracted genomic DNA was then stored at − 20 °C until analysis.

Polymerase chain reaction (PCR) primers and single-base extension primers were designed according to the Assay Design Suite V2.0 (Sequenom) online software (http://www.mysequenom.com). In this study, a total of 29 SNPs from four CYP450s were selected for genetic polymorphism analysis: CYP3A4 (rs28371759, rs4646440, rs4646437, rs3735451, rs2246709, and rs2242480); CYP3A5 (rs1419745, rs4646450, rs15524, rs776746, and rs3800959); CYP2C8 (rs2071426, rs17110453, rs1341159, rs1557044, rs10772526, rs6583969, rs11572139, rs7909236, rs2185571, rs1934952, rs11572162, and rs1058932); and CYP2D6 (rs28371699, rs4078247, rs28670611, rs1080983, rs35028622, and rs5758589). SNP typing was completed on a MassARRAY system (Sequenom), which was based on matrix-assisted laser desorption ionization time-of-flight mass spectrometry (MALDI-TOF). The PCR amplification conditions were as follows: pre-denaturation at 95 °C for 5 min; followed by 25 cycles of denaturation at 95 °C for 30 s, annealing at 58 °C for 30 s, extension at 72 °C for 1 min; and a final extension at 72 °C for 5 min. The final results were genotyped using the MassARRAY Typer 4.0 software system (Sequenom Inc., San Diego, CA, USA).

### Statistical analysis

Data were analysed using IBM SPSS Statistics (version 26, IBM Corporation, Armonk, NY, USA). Quantitative data were presented as mean and standard deviation, while qualitative data were presented as number and percentage. The chi-square test was used to compare the Hardy–Weinberg equilibria and adverse reactions of patients with different genotypes, and T-tests and one-way analysis of variance were used to compare the blood concentration of HCQ among groups. *P* < 0.05 was considered to indicate statistical significance.

## Results

### Patient characteristics

**Fig. 1:**
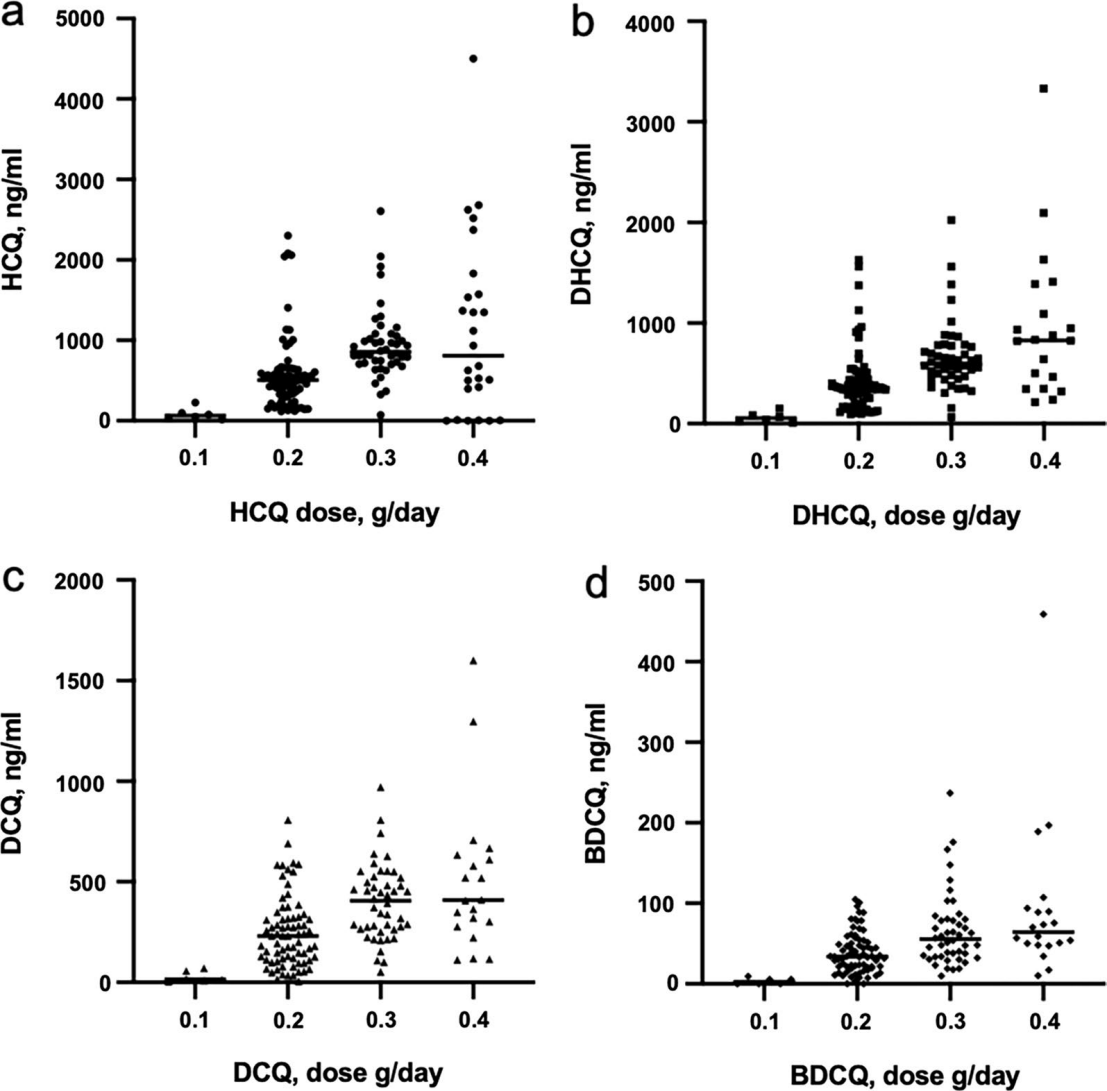
Correlation between the daily dose groups and concentrations of HCQ and its metabolites. **a** Concentration of hydroxychloroquine (HCQ); **b** concentration of desethyl hydroxychloroquine (DHCQ); **c** concentration of desethyl chloroquine (DCQ); **d** concentration of bisdesethyl chloroquine (BDCQ)

**Fig. 2:**
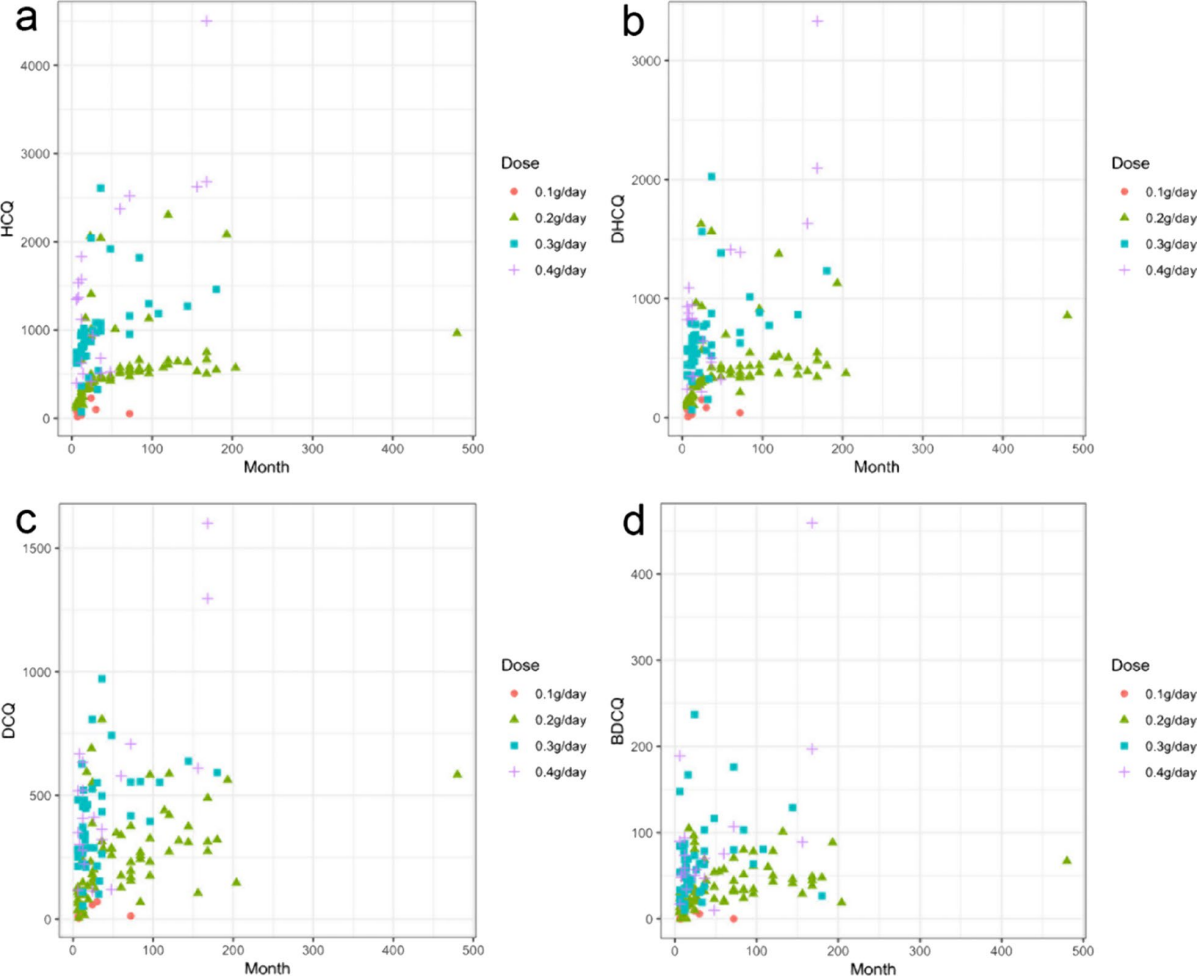
Time-course of blood concentrations of HCQ and its metabolites in SLE and RA patients receiving 100, 200, 300 or 400 mg HCQ daily. **a** HCQ; **b** DHCQ; **c** DCQ; and **d** BDCQ (n = 6, n = 74, n = 46 and n = 20 for 100, 200, 300 and 400 mg dose groups, respectively.)

A total of 146 participants (n = 121 SLE and n = 25 RA; 7 male and 139 female; mean age: 42.27 ± 14.13 years) with pathologic diagnosis were enrolled. The characteristics of the patients are shown in Table 1. The mean duration of oral HCQ was 51.70 ± 46.50 months, and the mean daily dose of HCQ was 255.48 ± 77.93 mg/day. The mean disease activity score 28 (DSA28) score of RA patients was 2.16 ± 0.41, and the mean SLEDAI score of SLE patients was 0.89 ± 1.81, which suggests that most participants had mild disease (DSA28 < 3.2 and SLEDAI < 9). The plasma concentrations of HCQ, DHCQ, DCQ, and BDCQ were 838.10 ± 522.00, 582.80 ± 363.90, 346.90 ± 205.30, and 56.00 ± 39.30 ng/ml, respectively. Grouped according to the dosage of patients, the results showed that blood concentrations of HCQ, DHCQ, DCQ, and BDCQ were significantly associated with the daily HCQ dosage (*P* < 0.005) (Fig. 1). The relationships between blood concentrations of HCQ, DHCQ, DCQ, and BDCQ and the duration of oral HCQ are shown in Fig. 2a–d, respectively. The blood concentrations of HCQ, DHCQ, DCQ, and BDCQ also showed a significant correlation (*P* < 0.005). The distribution of ADRs among all patients was as follows: abnormal renal function (n = 21), abnormal liver function (n = 11), ophthalmic ADRs (n = 20), and skin and mucous membrane ADRs (n = 15).

**Table 1:** Characteristics of the study participants

Characteristic	No. of patients, N = 146
SLE, no. (%)	121 (82.80)
RA, no. (%)	25 (17.20)
Age, mean ± SD years	42.27 ± 14.13
Female, no. (%)	139 (95.20)
Weight, mean ± SD kg	56.89 ± 9.21
Duration of HCQ treatment, mean ± SD months	51.70 ± 46.50
HCQ dose, mean ± SD mg/day	255.48 ± 77.93
HCQ dose, mean ± SD mg/kg/day	4.58 ± 1.50
Daily prednisolone dose, mean ± SD mg	4.77 ± 4.33
DSA28 score, mean ± SD	2.16 ± 0.41
SLEDAI score, mean ± SD	0.89 ± 1.81
[HCQ], mean ± SD ng/ml	838.10 ± 522.00
[DHCQ], mean ± SD ng/ml	582.80 ± 363.90
[DCQ], mean ± SD ng/ml	346.90 ± 205.30
[BDCQ], mean ± SD ng/ml	56.00 ± 39.30
*ADR, no. (%)*	
Abnormal renal function	21 (14.30)
Abnormal liver function	11 (7.50)
Ophthalmic ADRs	20 (13.70)
Skin and mucous membrane ADRs	15 (10.30)

SLEDAI, Systemic Lupus Erythematosus Disease Activity Index; ADRs, Adverse drug reactions; Disease activity score 28, DAS28; [HCQ], HCQ concentration; [DHCQ], Desethyl hydroxychloroquine concentration; [DCQ], Desethyl chloroquine concentration; [BDCQ], Bisdesethyl chloroquine concentration

### Hardy–Weinberg equilibrium (HWE)

The genotype distribution based on HWE is shown in Table 2. The genotyping results of the 29 SNPs in 146 SLE and RA patients showed that the allele frequencies of the 23 SNPs were consistent with HWE (*P* > 0.05), and the observed and expected values of alleles and genotypes showed good agreement, which indicates that the samples included in this study were representative of the population. CYP 2C8 (rs1341159) and CYP2D6 (rs28371699, rs4078247, rs35028622, rs28670611, and rs1080983) were not found other mutations (*P* < 0.05); therefore, no statistical analysis was required.

**Table 2:** HWE test of genotypes in 146 patients

Polymorphism	SNP	HWE *P*-value	Polymorphism	SNP	HWE *P*-value
CYP 2C8	rs2071426	0.23758	CYP3A4	rs28371759	0.93359
	rs17110453	0.09269		rs4646440	0.29517
	rs1341159	0.04798*		rs4646437	0.75722
	rs1557044	0.18561		rs3735451	0.85980
	rs10882526	0.69981		rs2246709	0.54361
	rs6583969	0.39241		rs2242480	0.12091
	rs11572139	0.40038	CYP2D6	rs28371699	0.00160*
	rs7909236	0.34911		rs4078247	0.00947*
	rs2185571	0.40038		rs28670611	0.00235*
	rs1934952	0.25790		rs1080983	0.00144*
	rs11572162	0.54993		rs35028622	0.00147*
	rs1058932	0.79740		rs5758589	0.07308
CYP3A5	rs1419745	0.91067			
	rs4646450	0.95620			
	rs15524	0.61950			
	rs776746	0.77629			
	rs3800959	0.43860			

^*^P < 0.05, indicates no genetic mutation and no statistical analysis is needed

### Relationship between CYP450 gene polymorphisms and ADRs

Samples from 146 patients with SLE and RA were used to analyse the association of SNPs with the risk of ADRs. In the genotype distribution analysis, CYP2C8 (rs1058932 and rs10882526), CYP3A5 (rs776746), and CYP3A4 (rs3735451) were related to ADRs. The frequencies of these polymorphisms are shown in Table 3. The ADRs of patients with CYP2C8 (rs1058932), CYP2C8 (rs10882526) and CYP3A5 (rs776746) genotype are shown in Table 4. Only 21 patients with abnormal renal function were included in the analysis of SNPs. Abnormal renal function was more prevalent in the AG genotype than with the AA genotype and GG genotype of CYP2C8 (rs1058932) (*P* = 0.017), while no significant differences between the different genotypes of CYP3A4 and CYP3A5 were noted (*P* > 0.05). CYP2C8 (rs10882526) is related to ophthalmic ADRs; the risk of the AA genotype was greater than that of the GG and AG genotypes (*P* = 0.026), while different genotypes of CYP3A4 and CYP3A5 showed no significant difference between normal and abnormal groups (*P* > 0.05). CYP3A5 (rs776746) was associated with the incidence of skin and mucous membrane ADRs in our research (*P* = 0.033), and the risk of the TT genotype was greater than that of the CC and CT genotypes; no significant difference was noted between the normal and abnormal skin and mucous membrane ADRs in different genotypes of CYP3A4 and CYP2C8 (*P* > 0.05). For the incidence rate of abnormal liver function, there was no significant difference for selected SNPs (*P* > 0.05).

**Table 3:** Frequencies of CYP 2C8, 3A5 and 3A4 polymorphisms

	Oligonucleotide primer	Allele frequency	Genotype	n (%)	HWE *P*-value
CYP2C8 (rs1058932)	r 5′-CTAGCCCATCTGGCTGC-3′	A = 35.3%	A/A	23 (15.8)	0.79740
		G = 64.7%	A/G	57 (39.0)	
			G/G	66 (45.2)	
CYP2C8 (rs10882526)	f 5′-TCAACTCACTCCGCT-3′	A = 87.0%	G/G	3 (2.1)	0.69981
		G = 13.0%	A/A	111 (76)	
			A/G	32 (21.9)	
CYP3A5 (rs776746)	f 5′-TCCAAACAGGGAAGAGATA-3′	C = 79.1%	C/T	55 (37.7)	0.77629
		T = 20.9%	T/T	8 (5.5)	
			C/C	83 (56.8)	
CYP3A4 (rs3735451)	f5′-AACAGAGTGATATTCTGATCTC-3′	C = 26.7%	C/C	10 (6.9)	0.85980
		T = 73.3%	C/T	58 (39.7)	
			T/T	78 (53.4)	

Values are the number (%)

**Table 4:** ADRs of patients with CYP2C8 (rs1058932), CYP2C8 (rs10882526), and CYP3A5 (rs776746)

ADR	Gene	SNP	Genotype	Normal group (n)	Abnormal group (n)	*P*	95% CI
Renal function	CYP2C8	rs1058932	AA	22	1	0.017	0.014–0.019
			AG	43	14		
			GG	60	6		
Ophthalmic	CYP2C8	rs10882526	GG	1	2	0.026	0.006–0.835
			AA	97	14		
			AG	28	4		
Skin and mucous membrane	CYP3A5	rs776746	CT	50	5	0.033	0.038–0.046
			TT	5	3		
			CC	36	7		

### Relationship between CYP450 gene polymorphisms and blood concentrations of HCQ and its metabolites

**Fig. 3:**
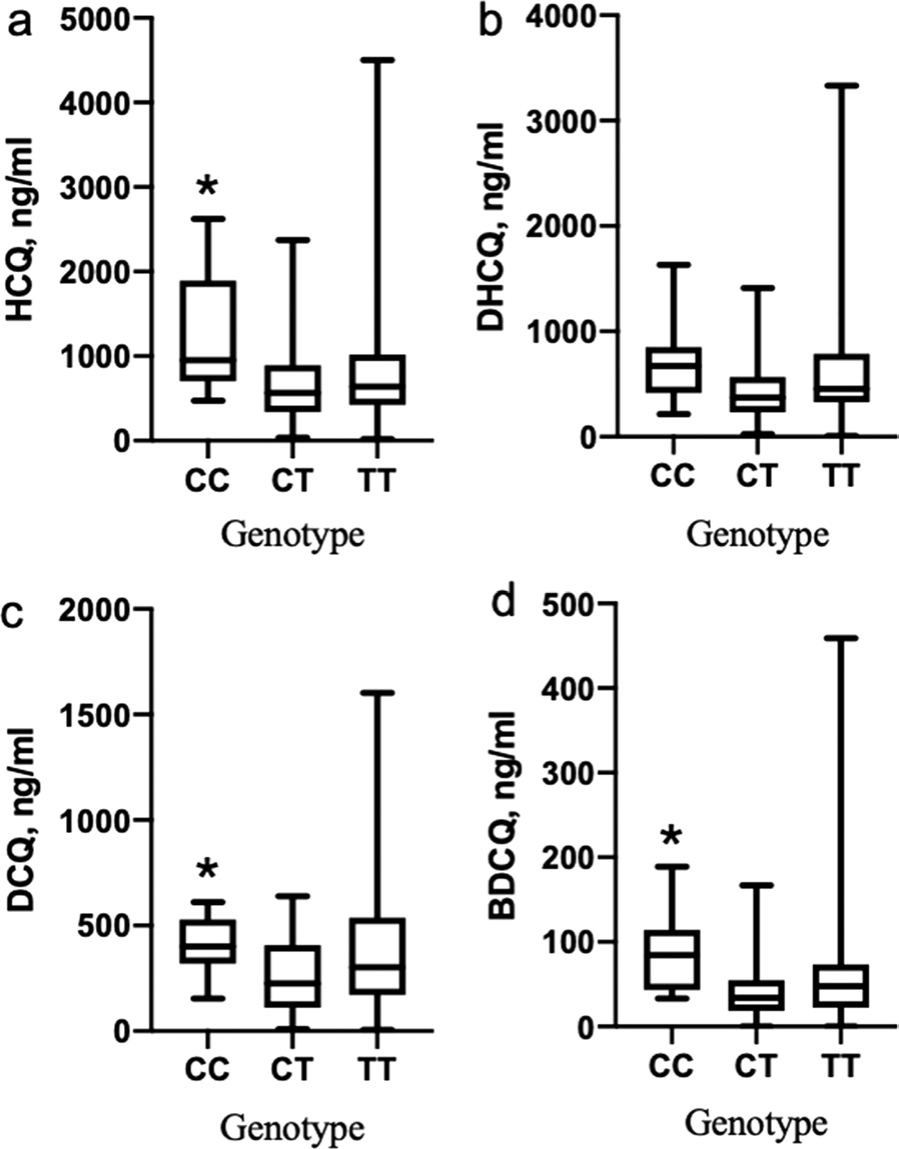
The distribution of HCQ blood concentration in different genotype groups in the adjusted model. **a** The distribution of HCQ in different genotype groups. The CC genotype group showed significantly higher concentration than the CT genotype group (*P* < 0.05), but no significant difference in the CT, TT genotype groups (*P* > 0.05). **b** The distribution of DHCQ in different genotype groups. There was no significant difference in the CC, CT and TT genotype groups (*P* > 0.05). **c** The distribution of DCQ in different genotype groups. The CC genotype group showed significantly higher concentrations than the CT genotype group (*P* < 0.05), but there was no significant difference in the CT and TT genotype groups (*P* > 0.05). **d** The distribution of BDCQ in different genotype groups. The CC genotype group showed significantly higher concentrations than the CT genotype groups (*P* < 0.05), but there was no significant difference in the CT and TT genotype groups (*P* > 0.05)

In the unadjusted model (patients taking medication for ≤ 10 years), only the CYP3A4 (rs3735451) polymorphism showed significant differences in the blood concentrations of HCQ, DCQ, and BDCQ (*P* = 0.033, *P* = 0.039, and *P* = 0.033). The mean blood concentrations of HCQ in SLE and RA patients with different CYP3A4 (rs3735451) genotypes are shown in Table 5. The HCQ, DCQ, and BDCQ concentrations were higher in patients with the CC genotype than those with the CT genotype. After adjusting for HCQ medication time (patients taking medication for > 10 years), this significant correlation still existed. For HCQ, DCQ, and BDCQ, the comparison between the CC and CT genotypes in patients was statistically significant (*P* < 0.05) (Fig. 3). The relationship between the genotypes of CYP3A4 (rs3735451) and the mean blood concentrations of HCQ, DHCQ, and DCQ were TT > CT > CC, while the CC genotype showed lower concentration than the other two genotypes of BDCQ.

**Table 5:** Results of average blood concentrations of HCQ and its metabolites in SLE and RA patients with different CYP3A4 genotypes

	SNP	Group	Genotype	n	Blood concentration (ng/ml)	*P*
Unadjusted model	rs3735451	HCQ	CC	8	742.4 ± 542.6*	0.033
			CT	53	723.8 ± 532.9	
			TT	69	742.1 ± 543.9	
		DHCQ	CC	8	512.3 ± 365.4	
			CT	53	500.1 ± 358.1	
			TT	69	512.1 ± 367.4	
		DCQ	CC	8	298.0 ± 200.3*	0.039
			CT	53	293.3 ± 198.8	
			TT	69	299.5 ± 202.9	
		BDCQ	CC	8	48.9 ± 39.2*	0.033
			CT	53	48.0 ± 38.5	
			TT	69	48.8 ± 39.0	
Adjusted Model^#^	rs3735451	HCQ	CC	10	759.9 ± 569.6*	0.017
			CT	58	795.9 ± 651.9	
			TT	78	797.0 ± 651.9	
		DHCQ	CC	10	519.8 ± 381.6	
			CT	58	553.5 ± 452.8	
			TT	78	554.1 ± 454.0	
		DCQ	CC	10	305.6 ± 271.3*	0.047
			CT	58	322.3 ± 239.3	
			TT	78	322.7 ± 241.0	
		BDCQ	CC	10	49.7 ± 40.4*	0.005
			CT	58	53.5 ± 52.5	
			TT	78	53.3 ± 52.4	

Note: vs. CT genotype, *P < 0.05 #: Adjusted for duration of use

## Discussion

HCQ is a hydroxyl derivative of chloroquine (CQ) and has a similar antimalarial activity to CQ. However, because HCQ has low toxicity, it can be used in the long-term treatment of SLE and RA. The plasma elimination half-life of HCQ is about 40–60 days, with higher tissue distribution and longer residence time in vivo [18]. Despite the potential efficacy and relative safety, the indications for and dosage of HCQ should be strictly controlled in clinical treatment. Furthermore, the ADRs should be closely observed, and HCQ blood levels of patients should be measured at regular intervals if necessary [19].

Studies have shown that there is a positive correlation between the efficacy of HCQ and its blood concentration. HCQ shows large inter-individual variations in its concentration, even in individuals taking the same dose [10]. About 40% of patients are unresponsive or intolerant to HCQ [20]. HCQ is mainly deacetylated by CYP450 enzymes 2D6, 2C8, and CYP3A4/5 [11, 12]; therefore, genetic polymorphisms of HCQ metabolic enzymes may be an important factor that affects individual concentration differences. Lee et al. [12] studied the effect of CYP2D6 gene polymorphisms on the blood concentration of HCQ in patients with SLE. They found a significant correlation between rs1065852 and rs1135840 and the ratio of DHCQ:HCQ, which indicates that the blood concentration of HCQ is related to the CYP2D6 gene polymorphism, but no similar results were found in CYP3A5*3 (rs776746) and CYP3A4*18B (rs28371759). Although some studies have reported that there was no significant correlation between CYP2C8 gene polymorphism and patients’ response to HCQ [10], our previous research showed that SLE patients with the CYP2C8 (rs10882521) GT genotype who took the same dose of HCQ had lower blood concentration than those with other genotypes, indicating that this SNP is related to the blood concentration of HCQ [21].

CYP3A4 is the most abundant CYP450 enzyme in the human liver, accounting for about 80% of the total CYP450 enzymes. The newly discovered CYP3A4*1G (rs2242480) is the site with the highest mutation frequency in the CYP3A4 allele, and the CYP3A4*1G gene polymorphism can reduce the catalytic activity of the CYP3A4 enzyme [22]. In our present study, a correlation between CYP3A4 (rs28371759) and (rs2242480) gene polymorphism and blood concentration of HCQ was not found, which was consistent with the results reported in the literature [12]. We found that CYP3A4 (rs3735451) was significantly correlated with blood concentration of HCQ and its metabolites by adjusting for the time of administration, and the mean blood concentrations of HCQ, DHCQ, and DCQ in patients with CC, CT, and TT genotypes is higher than those of other genotypes, with the blood concentration of HCQ and its main metabolite DHCQ being the lowest for the CC genotype. It is suggested that for patients with CYP3A4 (rs3735451) site mutation, a higher dose may be needed in clinical treatment to achieve an effective blood concentration and therapeutic effect.

Common ADRs are listed in the manual of HCQ sulfate tablets, including vision, skin, gastrointestinal tract, central nervous system, neuromuscular, cardiovascular system, haematological, liver, and other allergic reactions [6, 23]. In fact, Munster et al. [10] found a correlation between gastrointestinal adverse events and elevated blood HCQ levels, and a potential relationship between ophthalmic adverse events and BDCQ levels. The incidence of ophthalmic ADRs in the previous literature was 7.5% [24]; the incidence in our present study was 14.4%, significantly higher than that previously reported. This may be due to the inclusion of more ophthalmic ADRs in our study, such as eye swelling, hyperaemia, blurred vision, and conscious ametropia. However, until now, there have been only a few reports on the association between ADRs of HCQ and CYP450 gene polymorphisms. In this study, we found a significant difference in the distribution of the CYP2C8 (rs10882526) GG genotype and AA + AG genotypes in the ophthalmic ADR group (*P* < 0.05), and the incidence of ADRs for the GG genotype was higher than that of the AA + AG genotypes. The most important predictors of ophthalmic ADRs in patients are high doses and long-term use of HCQ; hence, mandatory ophthalmic evaluation should be performed during long-term use of HCQ to ensure drug safety [25].

HCQ is eventually excreted via the kidney. The reduced clearance in patients with renal insufficiency leads to HCQ accumulation in the body, significantly increasing the blood concentration after medication and the risk of drug poisoning. However, the dosage of HCQ in patients with renal insufficiency is not clear [26]. In this study, we analysed the relationship between CYP2C8 gene polymorphism and renal dysfunction. This showed a significant difference in the distribution of the AG genotype and AA + GG genotypes of CYP2C8 (rs1058932) between the normal and abnormal renal function groups. In addition, the distribution of the CYP3A5 (rs776746) TT genotype and CT + CC genotypes in patients with long-term HCQ use was significantly different from that in the normal group (*P* < 0.05). There are few reports about liver injury caused by HCQ; these mainly include liver drug enzyme abnormality and acute liver failure [27, 28]. Abnormal liver function, manifested by liver enzyme increase (aspartate aminotransferase, AST), was found in 11 patients in our study, but no influence of CYP450 gene polymorphism was found. A possible reason for this was that we only took the dose and course of treatment into account, and the combined medication was not considered. These results suggest that patients should regularly monitor their liver function during HCQ treatment and should reduce the dose or stop medication if necessary.

An earlier study showed that the DHCQ: HCQ ratio was associated with CYP2D6 (rs1065852 and rs1135840) polymorphisms after taking oral HCQ [12]. Our result was not consistent with that from a previous study, and we could not examine this polymorphism in our study because the frequency of CYP2D6 polymorphisms was confirmed to be extremely low in our study population (based on HWE analysis). Another possible limitation is the limited sample size in our study. Still, further studies that relate gene polymorphisms to HCQ metabolism and ADRs are needed to confirm our findings and discover more promising candidate SNPs.

The gene polymorphisms of the CYP enzyme may be important determinant of drug sensitivity and ADRs among different individuals. In the clinic, patients with the CYP2C8 (rs1058932) AG genotype had a higher incidence of renal dysfunction than those with the AA and GG genotypes after taking HCQ. Patients with the CYP2C8 (rs10882526) GG genotype had a higher incidence of ophthalmic ADRs than AA genotype and AG genotypes after taking HCQ. In addition, patients with the CYP3A5 (rs776746) TT genotype had a higher incidence of skin and mucosal ADRs than the CC and CT genotypes after taking HCQ. Given the same dose of HCQ, the blood concentration of HCQ in patients with the CYP3A4 (rs3735451) CC genotype was lower than that in patients with other genotypes. Thus, it may be helpful to improve the efficacy and reduce the ADRs by genotyping the relevant CYP450 gene polymorphisms before administration of HCQ and monitoring the concentration of HCQ and its metabolites. Physicians should try to offer individualised treatment with HCQ based on the results of CYP2C8 (rs1058932 and rs10882526), CYP3A4 (rs3735451), and CYP3A5 (rs776746) genotyping and HCQ blood concentration.

## Data Availability

The datasets used and analysed in this study are available from the corresponding author on reasonable request.

## Abbreviations

HCQ: Hydroxychloroquine
SLE: Systemic lupus erythematosus
RA: Rheumatoid arthritis
CYP 450: Cytochrome P450
ADRs: Adverse drug reactions
SNPs: Single nucleotide polymorphisms
GCs: Glucocorticoids
DMARDs: Disease-modifying anti-rheumatic drugs
SLEDAI: SLE disease activity index
DHCQ: Desethyl hydroxychloroquine
DCQ: Desethyl chloroquine
LC–MS/MS: Liquid chromatography-tandem mass spectrometry
BDCQ: Bisdesethyl chloroquine
CQ: Chloroquine
HPLC: High-performance liquid chromatography
HWE: Hardy–Weinberg equilibrium
EULAR: European League Against Rheumatism
